# Supplementary material for: Optical inter-site spin transfer probed by energy and spin-resolved transient absorption spectroscopy
Source: Nat Commun. 2020 Feb 13;11:871. doi: 10.1038/s41467-020-14691-5 (PMC7018696; doi:10.1038/s41467-020-14691-5)
Supplement: Supplementary file 1 — Supplementary Information [file 41467_2020_14691_MOESM1_ESM.pdf]

OPTICAL INTER-SITE SPIN TRANSFER PROBED BY ENERGY AND SPIN-RESOLVED TRANSIENT ABSORPTION SPECTROSCOPY

Felix Willems[1], Clemens von Korff Schmising[1*], Christian Strüber[1], Daniel Schick[1], Dieter W. Engel[1], J. K. Dewhurst[2], Peter Elliot[1], Sangeeta Sharma[1] and Stefan Eisebitt[1,3]

[1]*Max Born Institute for Nonlinear Optics and Short Pulse Spectroscopy, Max-Born-Strasse 2A, 12489 Berlin, Germany*
[2]*Max-Planck-Institute for Microstructure Physics, Weinberg 2, 06120 Halle (Saale), Germany*
[3]*Institut für Optik und Atomare Physik, Technische Universität Berlin, 10623 Berlin, Germany*

*korff@mbi-berlin.de

Supplementary Discussion

Energy-dependent magnetic asymmetry and demagnetization constants. Figure 1 displays the absolute magnetic asymmetry in the time interval between -1 and 2 ps. For the CoPt alloy we show data for all measured photon energies, for the Co film we only show the response at the M-edge resonance at 60.3 eV. Note that because a non-negligible remagnetization the data has to be described by a double exponential function to correctly determine the respective time constants.

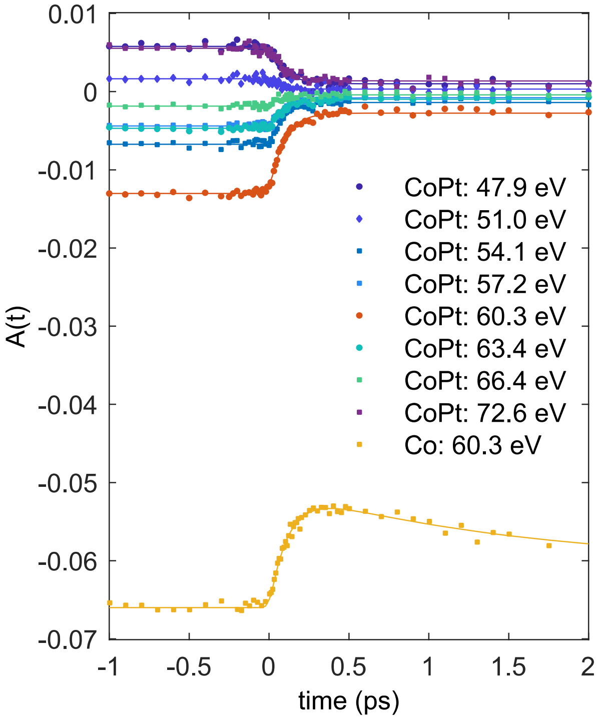

Supplementary Figure 1| Time resolved magnetic asymmetry. Measured absolute asymmetry, A(t), in a time interval between -1 and 2 ps. For the CoPt alloy we show data for all measured photon energies, for the Co film only at the M-edge resonance at 60.3 eV. The solid lines correspond to a non-linear least square fit.

The data is fitted with Eq. 4 of the main text and the result is shown as solid lines in Fig. 1. Table 1 displays the retrieved static magnetic asymmetries, $A(t<t_0)$, the relative demagnetization amplitudes, $M_1/A(t<0)$, and demagnetization time constants, τ_D, for the Co and CoPt sample measured at different photon energies, including the Pt O_3 (54.1eV, 57.1 eV), Co $M_{2,3}$ (60.3 eV) and Pt N_7 (72.6 eV) resonance. The measured values of $A(t<0)$ are in good agreement with static characterization measurements carried out at the synchrotron facility BESSYII, HZB [1-2]. Note that even for small values of $A(t<t_0)$ on the order of only a few permille, measurements still yield parameters with very small uncertainties. Within the experimental error, the demagnetization amplitudes and time constants of $Co_{0.5}Pt_{0.5}$ are the same for all photon energies and yield an average value of $\overline{M_1/A\,(t<0)} = (0.79 \pm 0.02)$ and $\overline{\tau_D} = (86 \pm 10)$ fs, i.e., show no element specificity. For the Co film the demagnetization constant at the Co $M_{2,3}$ (60.3 eV), however, is significantly larger, $\tau_D = (124 \pm 4)$ fs while the relative demagnetization amplitude is only (0.24 ± 0.01). Remagnetization takes place on a much longer time scale is therefore not relevant for the discussion in the present paper. All stated errors correspond to an uncertainty interval of σ (68%).

	CoPt									Co
			Pt O_3	Pt O_3	Co $M_{2,3}$				Pt N_7	Co $M_{2,3}$
E [eV]	47.9	50.1	54.1	57.1	60.3	63.4	66.4	69.5	72.6	60.3
A(*t*<0) [%]	0.58± 0.008	0.16± 0.007	-0.67± 0.007	-0.44± 0.006	-1.3± 0.006	-0.47± 0.005	-0.18± 0.005	0	0.55± 0.007	-6.6± 0.007
τ_D [fs]	90±10	77±27	84±7	103±11	86±3	88±8	69±14	/	92±11	124±4
M_1/A(t<0)	-0.83± 0.03	-0.80± 0.09	-0.79± 0.02	0.82± 0.03	-0.79± 0.01	-0.79± 0.02	-0.76± 0.05	/	-0.75± 0.03	-0.24± 0.01

Supplementary Table 1| Fitting parameters of the demagnetization. Static magnetic asymmetries, *A*(*t*<0), relative demagnetization amplitudes, M_1/A(t<0), and demagnetization constants, τ_D, for the CoPt alloy and the pure Co film measured at selected photon energies, including the Pt O_3 (54.1eV, 57.1 eV), Co $M_{2,3}$ (60.3 eV) and Pt N_7 (72.6 eV) resonance.

Loss of minority electrons in Pt. In Fig. 2 we show the calculated, normalized sum of occupied minority and majority d-states, $n_d(t,E)$, integrated over an energy range extending from -13 eV to 13 eV as a function of pump-probe delay:

$$\int \Sigma n_d(t,E)dE = \int_{-13\mathrm{eV}}^{+13\mathrm{eV}} \left(n_{d,min}(t,E) + n_{d,maj}(t,E)\right) dE$$

Since the total charge is conserved, this quantity remains constant in the Co film after laser excitation, i.e., the loss of majority states is compensated by a loss of minority states. This simply restates the results already shown in the inset of Fig. 3c in the main text. Charge conservation in the CoPt alloy requires that the experimentally and theoretically observed gain of minority electrons in Co is compensated by a loss of minority electrons in Pt. This is exactly what we observe in Fig. 2: the loss of majority electrons due to efficient spin-flip processes does not equal the gain in minority electrons; instead, they are efficiently transferred from Pt to empty Co states.

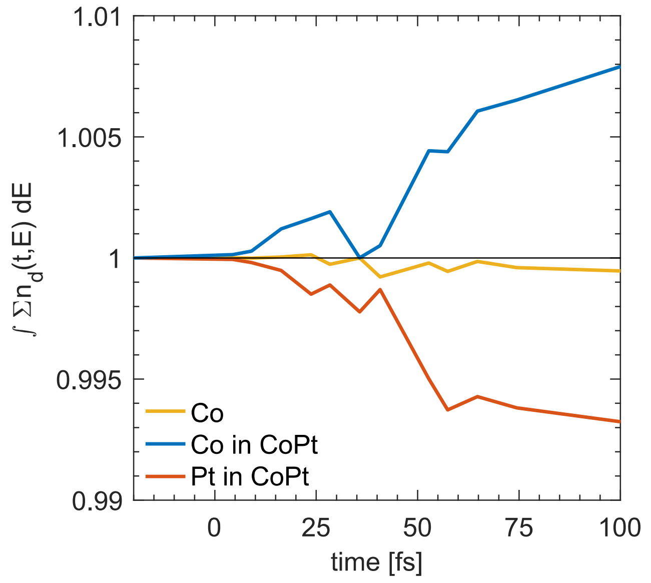

Supplementary Figure 2| Element specific charge flow of d-electrons. Energy integration over the sum of occupied minority and majority *d*-electrons, $n_d(t,E)$, for Co as well as for Co and Pt in the CoPt alloy. Because the total charge is conserved, the gain in minority states of Co is compensated by a loss of minority states of Pt in CoPt. This directly corroborates our hypothesis that in CoPt, we have an inter-site spin transfer of minority electrons from Pt 5*d* to Co 3*d* states.

Supplementary References

[1]Dewhurst, J. K. *et al.* Element specificity of transient extreme ultra-violet magnetic dichroism. Preprint at https://arxiv.org/abs/1909.00199 (2019).

[2] Willems, F. *et al.* Magneto-Optical Functions at the 3 p Resonances of Fe, Co, and Ni: Ab initio Description and Experiment. *Phys. Rev. Lett.* **122**, 217202 (2019).
